# Ovarian Hyperstimulation Syndrome: An Unusual Cause of Abdominal Pain Presenting to the Emergency Department

**DOI:** 10.7759/cureus.111397

**Published:** 2026-06-23

**Authors:** Atira Shenoy, Rahul Ramakrishnan, Malek F Amro, Alejandro Biglione

**Affiliations:** 1 Medical Education, Nova Southeastern University Dr. Kiran C. Patel College of Osteopathic Medicine, Fort Lauderdale, USA; 2 Medical Education, Nova Southeastern University Dr. Kiran C. Patel College of Allopathic Medicine, Fort Lauderdale, USA; 3 Internal Medicine, Wellington Regional Medical Center, Wellington, USA

**Keywords:** abdominal emergency medicine, abdominal pain in females, assisted reproductive technology (art), controlled ovarian hyperstimulation, ovarian hyperstimulation syndrome (ohss)

## Abstract

The use of assisted reproductive technologies (ART), such as in vitro fertilization (IVF), is steadily increasing. Ovarian hyperstimulation syndrome (OHSS) is a rare but potentially serious complication arising from controlled ovarian hyperstimulation (COH), a critical component of ART. OHSS may vary in severity and usually causes abdominal pain, nausea, vomiting, diarrhea, and dyspnea, which are common complaints for patients presenting to the emergency department (ED). Preventive measures and early recognition by reproductive medicine specialists generally reduce the incidence of severe OHSS, making ED presentations uncommon. However, patients may present to the ED and require the ED physician to provide initial evaluation and management. The patients often require admission to the hospital and may be cared for by physicians who are not reproductive medicine specialists. This case report describes a 35-year-old woman who developed severe abdominal pain following oocyte retrieval and oocyte pick-up at a fertility clinic and subsequently presented to the ED for evaluation. The report highlights the diagnosis and management of OHSS, incorporating current literature and guideline recommendations, particularly for non-reproductive medicine specialists, such as ED physicians and hospitalists, who may need to care for patients with this complication.

## Introduction

Infertility, defined as the inability to achieve a successful pregnancy after 12 months of regular unprotected intercourse, affects approximately 17.5% of the adult population [1-2]. Assisted reproductive technologies (ART), including in vitro fertilization (IVF), are being increasingly utilized in the treatment of infertility. Controlled ovarian hyperstimulation (COH), a key component of ART, promotes the development of multiple follicles through the administration of exogenous gonadotropins and ovulation-induction protocols [3].

COH protocols are based on the administration of gonadotropins with follicle-stimulating hormone (FSH)-like activity, such as human menopausal gonadotropin (hMG) or recombinant FSH. COH requires strict monitoring to avoid an exaggerated ovarian response. COH protocols also require pharmacological measures to prevent a premature luteinizing hormone (LH) peak that would cause spontaneous ovulation, which would prevent the physician from retrieving the oocytes of the ovarian follicles via transvaginal puncture. The most common strategy for this purpose is to use a gonadotropin receptor hormone (GnRH) antagonist such as cetrorelix or ganirelix. The final maturation of the oocytes is achieved by using a trigger injection with preparations with LH-like action, such as human chorionic gonadotrophin (HCG), recombinant LH, or a GnRH agonists that produce an endogenous LH surge due to a flare-up effect. Mature oocytes are aspirated from the ovaries approximately 36 hours after the administration of the trigger injection. In intracytoplasmic sperm injection (ICSI), a single spermatozoa is injected into the cytoplasm of the oocyte. If the oocytes are successfully fertilized, they are typically incubated and monitored for successful development. The embryos are transferred into the uterus in a process called embryo transfer (ET). If ET takes place within the same cycle, it is called fresh ET. If embryos are not transferred in the present cycle, they can be cryopreserved to be transferred in another cycle, called frozen ET.

Although typically safe, COH may result in ovarian hyperstimulation syndrome (OHSS), a rare but potentially life-threatening iatrogenic complication. Although the overall incidence of severe OHSS is relatively low, ranging from 0.9% to 1.4%, the syndrome may present with a broad spectrum of clinical manifestations ranging from mild abdominal discomfort to life-threatening complications such as respiratory failure, renal dysfunction, and thromboembolism [4]. Mild OHSS is estimated to occur in 20-33% of IVF cycles, while moderate to severe OHSS occurs in about 1-5% of IVF cycles [5-6]. Evidence on the incidence of critical OHSS is not readily available.

Because OHSS may have overlapping features with other conditions, prompt recognition and management by the emergency department (ED) and primary care physicians are essential to reduce morbidity and mortality. This case report depicts a 35-year-old woman who developed severe abdominal pain following oocyte retrieval at an outside fertility clinic and sought evaluation at her local ED.

## Case presentation

A 35-year-old woman with a past medical history of polycystic ovarian syndrome (PCOS) and infertility presented to the ED with severe right upper quadrant and epigastric abdominal pain associated with nausea, vomiting, and diarrhea. The pain began one day before presentation and progressively worsened. She denied radiation of pain and reported no previous episodes of similar symptoms. The pain was described as “aching” and was rated 10/10. She did not attempt measures to alleviate the pain. 

The patient had recently undergone a cycle of COH for IVF using the following antagonist protocol: follitropin beta for 10 days to promote multifollicular development, followed by cetrorelix beginning on stimulation day 6 to prevent premature ovulation. Final oocyte maturation was induced with leuprolide, a GnRH agonist. She subsequently underwent an uneventful oocyte pick-up (OPU), during which 37 oocytes were retrieved. The retrieved oocytes underwent IVF, and the resulting embryos were vitrified for future frozen ET.

Upon presentation, her vital signs revealed a temperature of 36.5°C, blood pressure of 119/68 mmHg, heart rate of 105 beats per minute, respiratory rate of 16 breaths per minute, and oxygen saturation of 95% on room air. On physical examination, the patient was alert and oriented to person, place, and time. She appeared uncomfortable and was unable to lie comfortably in bed. No jugular venous distention was noted. Cardiac examination revealed a regular rate and rhythm without murmurs, rubs, or gallops. Respiratory examination was notable for decreased breath sounds at the right lung base, with no wheezes, rales, stridor, or rhonchi. The patient exhibited mildly increased work of breathing without tachypnea or accessory muscle use. Abdominal examination revealed a soft but distended abdomen with diffuse tenderness to palpation without guarding or rebound tenderness. There were no signs of peritonitis.

Laboratory results on admission and 13 days prior to admission are summarized in Table 1. Urinalysis on admission is summarized in Table 2. 

**Table 1:** Laboratory values on admission and 13 days prior

Lab value	Patient value (on admission)	Patient value (13 days prior)	Normal range
White blood cell count	14.42 x 10^3^/µL	6.71 x 10^3^/µL	4.50 x 10^3^/µL-10.50 x 10^3^/µL
Red blood cell count	3.74 x 10^6^/µL	4.31 x 10^6^/µL	4.40 x 10^6^/µL-6.15 x 10^6^/µL
Hemoglobin	10.7 g/dL	12 g/dL	14.0 g/dL-18.0 g/dL
Hematocrit	31.9%	36.8%	40.0%-54.0%
Mean corpuscular volume	85.3 fL	84.8 fL	81.0 fL-96.0 fL
Mean corpuscular hemoglobin	28.6 pg	28.1 pg	27.0 pg-34.0 pg
Platelet count	238 x 10^3^/µL	279 x 10^3^/µL	150 x 10^3^/µL-450 x 10^3^/µL
Prothrombin time	12.8 seconds	N/A	9.7 seconds-11.7 seconds
Glucose	113 mg/dL	86 mg/dL	74 mg/dL-106 mg/dL
Sodium	137 mmol/L	139 mmol/L	135 mmol/L-148 mmol/L
Potassium	4.3 mmol/L	3.8 mmol/L	3.6 mmol/L-5.2 mmol/L
Chloride	108 mmol/L	107 mmol/L	95 mmol/L-110 mmol/L
Bicarbonate	27 mEq/L	27 mEq/L	21 mEq/L-32 mEq/L
Blood urea nitrogen	13 mg/dL	14 mg/dL	7 mg/dL-18 mg/dL
Creatinine	0.80 mg/dL	0.93 mg/dL	0.7 mg/dL-1.3 mg/dL
Calcium	8.2 mg/dL	9.2 mg/dL	8.5 mg/dL-10.1 mg/dL
Albumin	2.2 g/dL	3.6 g/dL	3.4 g/dL-5.0 g/dL
Total bilirubin	0.2 mg/dL	0.3 mg/dL	0.0 mg/dL-1.0 mg/dL
HCG QT	6 mIU/mL	N/A	< 5 mIU/mL

**Table 2:** Urinalysis on admission HPF: high-power field

Urinary value	Patient value	Normal range
Color	Yellow	Yellow
Clarity	Cloudy	Clear
Specific gravity	1.023	1.005-1.025
pH	5	4.5-8
Leukocyte esterase	Moderate	Negative
Nitrite	Negative	Negative
Protein	Negative	≤150 mg/d
Glucose	Negative	≤130 mg/d
Ketones	Negative	Negative
Urobilinogen	Negative	0.5-1 mg/dL
Bilirubin	Negative	Negative
Blood	Negative	≤3 RBCs
White blood cell	4-10 WBCs/hpf	≤2-5 WBCs/hpf
Red blood cell	0 -3 RBCs/hpf	≤2 RBCs/hpf
Bacteria	2+	Absent
Squamous epithelium	11-20 squamous epithelial cells/hpf	≤15-20 squamous epithelial cells/hpf

Transvaginal ultrasound demonstrated bilaterally enlarged ovaries with multiple follicles, consistent with recent ovarian stimulation (Figure 1).

**Figure 1:**
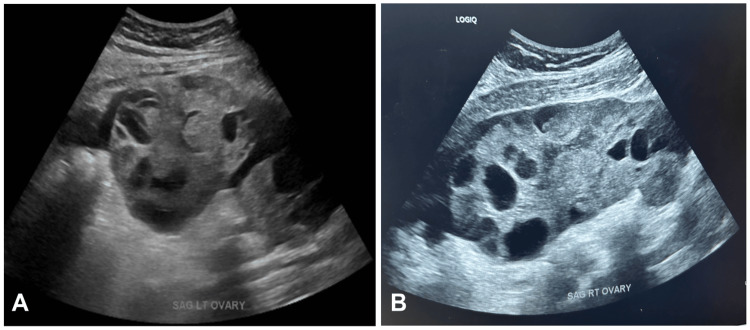
Transvaginal ultrasound This image depicts ovarian enlargement and numerous follicles in the left (A) and right (B) ovary of the patient.

Abdominal ultrasound revealed right upper quadrant intraperitoneal free fluid (Figure 2A) and a right-sided pleural effusion (Figure 2B).

**Figure 2:**
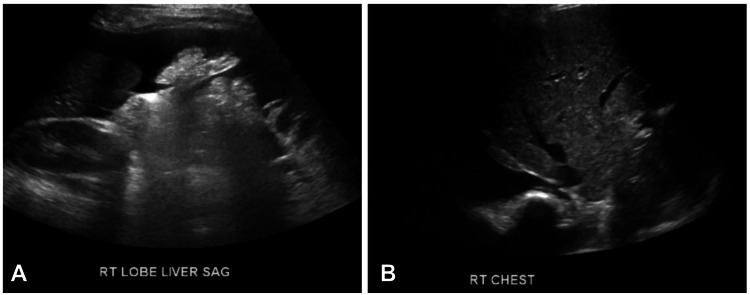
Abdominal ultrasound This image demonstrates right upper quadrant intraperitoneal free fluid (A) and a right-sided pleural effusion (B) in the patient.

Chest radiography confirmed the presence of a right pleural effusion (Figure 3).

**Figure 3:**
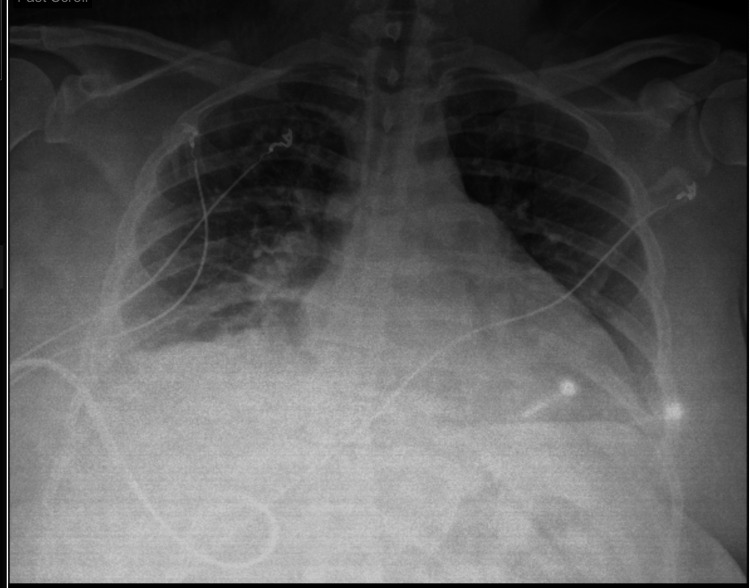
Chest X-ray consistent with right pleural effusion

A CT was ordered to further investigate the patient’s condition. The patient’s CT abdomen and pelvis with contrast demonstrated enlarged bilateral ovaries and multiple follicles (Figure 4).

**Figure 4:**
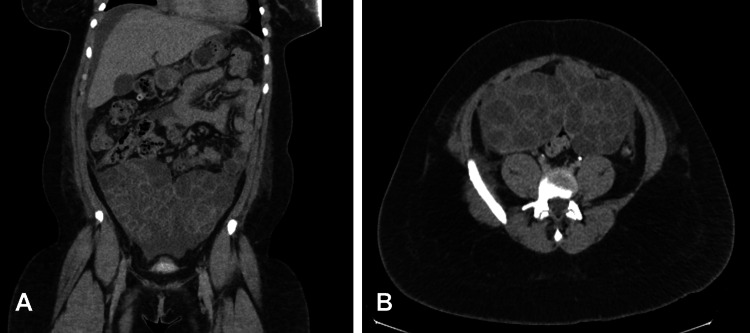
CT abdomen and pelvis with contrast These images depict bilateral ovaries enlarged in size and multiple follicles within coronal (A) and axial (B) views. CT: computed tomography

The patient was admitted with a diagnosis of severe OHSS complicated by ascites and right-sided pleural effusion. Obstetrics and Gynecology (OB/GYN) was consulted by the admitting hospitalist team. However, reproductive medicine services were not available at our institution.

Given concerns for intravascular volume depletion secondary to third spacing, she was started on lactated Ringer’s solution at 125 mL/hour, albumin 25% (50 g twice daily), cabergoline 0.5 mg daily, acetaminophen 1000 mg IV every 6 hours as needed, ondansetron 4 mg IV every 6 hours as needed, and venous thromboembolism (VTE) prophylaxis with enoxaparin 40 mg subcutaneously daily. Albumin was administered as an adjunctive treatment for severe OHSS as it helps restore plasma oncotic pressure and improve effective circulating volume in this condition. On hospital day 5, the patient reported significant symptomatic improvement and was discharged home in stable condition.

## Discussion

OHSS is a potentially life-threatening iatrogenic complication of COH characterized by ovarian enlargement, increased vascular permeability, and third-spacing of intravascular fluid. The pathophysiology of OHSS is multifactorial and is thought to involve three primary mechanisms [7]. First, there is enhanced capillary permeability that promotes extravascular fluid movement due to vascular endothelial growth factor (VEGF) release by granulosa cells [8-9]. Second, activation of the ovarian renin-angiotensin system (OVRAS) contributes to third-spacing of fluid through angiotensin overexpression and receptor activation [10-11]. Third, various signaling molecules, such as interleukin-1β, interleukin-6, angiotensin II, epidermal growth factor, insulin-like growth factor, and platelet-derived growth factor, contribute to the pathogenesis of OHSS [12]. In severe OHSS, these mechanisms result in a significant increase in vascular permeability, leading to substantial third-space fluid shifts, which may lead to massive ascites, pleural effusions, hemoconcentration, intravascular volume depletion, acute kidney injury, respiratory compromise, and thromboembolic events. These manifestations were shown in our patient, who developed significant intraperitoneal free fluid and a pleural effusion requiring hospitalization. 

OHSS is most commonly triggered by exposure to human chorionic gonadotropin (hCG), either administered exogenously to induce final oocyte maturation or produced endogenously during pregnancy. Spontaneous cases are rare and typically occur after a molar pregnancy or in individuals with mutations in the FSH receptor that cause hypersensitivity to stimulation. Iatrogenic OHSS is more common and is associated with exogenous hCG administration during ovarian hyperstimulation. 

OHSS is classified by etiology (spontaneous vs. iatrogenic), time of onset (early vs. late), and severity (mild, moderate, severe, critical). Early-onset OHSS typically develops within 10 days of hCG administration, while late-onset OHSS occurs beyond 10 days and is often associated with endogenous hCG production from pregnancy. The American Society for Reproductive Medicine (ASRM) categorizes OHSS severity as mild, moderate, severe, and critical. Mild disease is generally characterized by abdominal discomfort, nausea, vomiting, and enlarged ovaries without significant laboratory abnormalities. Moderate OHSS may include hemoconcentration, lymphocytosis, and mild ascites. Severe OHSS is associated with laboratory abnormalities, such as impaired creatinine clearance, hyperkalemia, and hyponatremia, along with worsening symptoms such as dyspnea and persistent vomiting. Critical OHSS is marked by rapid weight gain (> 1 kg in 24 hours), hypotension, acute renal failure, pericardial effusion, and respiratory distress syndrome [6,13].

Given the nonspecific presentation, differential diagnoses may include polycystic ovaries, ovarian torsion, ovarian cyst rupture, ectopic pregnancy, pelvic abscess, pelvic infection, nephrolithiasis, pulmonary embolism, mesenteric ischemia, bowel obstruction, cholecystitis, and appendicitis [14]. Although the patient presented with ascites and diffuse abdominal pain, spontaneous bacterial peritonitis (SBP) was less likely given the absence of fever, hemodynamic instability, and no evidence of intra-abdominal infection. The patient’s improvement without antibiotic therapy further excluded SBP from the differential, explaining why our team did not proceed with a diagnostic paracentesis. In this patient, the recent ovarian stimulation history combined with imaging findings of bilaterally enlarged multicystic ovaries, ascites, and pleural effusion supported the OHSS diagnosis.

The patient's laboratory findings were also consistent with the pathophysiology of OHSS. Leukocytosis may occur due to systemic inflammation and physiologic stress without an infection. The patient experienced marked hypoalbuminemia (2.2 g/dL) due to increased vascular permeability and third-spacing of protein-rich fluid into the peritoneal and pleural spaces. This finding further reinforced the use of albumin as supportive management. Although severe OHSS can be associated with hemoconcentration, our patient demonstrated mild anemia without clinical or radiographic evidence of active hemorrhage. Renal function remained stable throughout hospitalization, with normal creatinine levels despite fluid redistribution.

Management of OHSS depends on disease severity and may range from outpatient monitoring to intensive inpatient management [14]. Mild to moderate cases are generally treated conservatively with rest, hydration, and close monitoring of symptoms, urine output, and hematocrit to assess intravascular volume. Severe cases often require hospitalization for fluid and electrolyte monitoring, imaging to evaluate complications, and therapeutic paracentesis. More severe cases may necessitate intensive care unit (ICU) admission for respiratory support, intravenous fluid resuscitation, and careful fluid balance management. As patients with OHSS are at a higher risk of VTE due to systemic inflammation and fluid shifts, VTE prophylaxis is essential in hospitalized patients. Surgical intervention is rare but may be required for complications such as ovarian torsion or intra-abdominal bleeding. Intra-abdominal hemorrhage may occur secondary to rupture of enlarged hyperstimulated ovaries, hemorrhagic ovarian cysts, or post-oocyte retrieval. Clinicians should remain cautious in patients with worsening abdominal pain, hemodynamic instability, declining hemoglobin levels, or pertinent imaging findings. Pregnancy termination is considered only in extreme, life-threatening cases. Furthermore, it is important to note that nonsteroidal anti-inflammatory medications should not be used in patients with moderate to severe OHSS due to increased risk of acute kidney injury. Because OHSS may already cause third-spacing of fluid and intravascular volume depletions, NSAIDs can further reduce renal perfusion and exacerbate kidney injury due to lower effective circulating volume. Patients with OHSS are at an already higher risk of acute kidney injury due to this volume depletion.

Preventive strategies focus on individualized ovarian stimulation protocols with close monitoring using serum estradiol levels and transvaginal ultrasound [15]. Preventive measures begin with identifying patients at increased risk, including those of young age, low body mass index (BMI), PCOS, previous history of OHSS, elevated anti-Müllerian hormone (AMH) levels, elevated estradiol levels during stimulation, and high antral follicle count. Our patient had several recognized risk factors for OHSS, including PCOS and age younger than 35 years old.

High-risk patients are often managed with GnRH antagonist protocols, GnRH agonist trigger injections, reduced gonadotropin dosing, and freeze-all embryo strategies to minimize OHSS risk. Additional preventive strategies for OHSS include coasting, low-dose hCG, cycle cancellation, follicular aspiration, modified ET protocols, and the use of dopamine agonists such as cabergoline. Cabergoline is effective in reducing the incidence and severity of OHSS by inhibiting VEGF-mediated vascular permeability, which can subsequently limit fluid third-spacing [16].

## Conclusions

OHSS is an uncommon but potentially life-threatening complication of COH, particularly with the widespread use of modern preventative protocols. Although severe OHSS requiring ED evaluation or hospitalization is rare, prompt recognition and appropriate management by reproductive medicine specialists, OB/GYN providers, emergency physicians, and hospitalists are critical to reduce morbidity and mortality. Evaluation should include assessment for complications such as electrolyte abnormalities, acute kidney injury, thromboembolic events, and significant fluid third-spacing. In our patient, the imaging findings and laboratory abnormalities were consistent with severe OHSS and outlined the condition’s pathophysiology of increased vascular permeability. Early recognition led to appropriate management, including fluid resuscitation, albumin administration, thromboprophylaxis, symptomatic control, and OB/GYN consultation. 

This case highlights the importance of maintaining a high clinical suspicion for OHSS in patients presenting with abdominal pain, ascites, or respiratory symptoms following recent ovarian stimulation. Emergency physicians, OB/GYN providers, and hospitalists should remain familiar with the diagnosis and management of OHSS to ensure timely intervention and prevent serious complications.
